# Exploring the optimum approach to the use of CT densitometry in a randomised placebo-controlled study of augmentation therapy in alpha 1-antitrypsin deficiency

**DOI:** 10.1186/1465-9921-10-75

**Published:** 2009-08-13

**Authors:** David G Parr, Asger Dirksen, Eeva Piitulainen, Chunqin Deng, Marion Wencker, Robert A Stockley

**Affiliations:** 1 Department of Respiratory Medicine, University Hospitals of Coventry and Warwickshire, Clifford Bridge Road, Coventry CV2 2DX, UK; 2 Gentofte Hospital, Copenhagen University, DK-2900 Hellerup, Denmark; 3 Department of Respiratory Medicine, University Hospital, Malmö, Sweden; 4 Talecris Biotherapeutics Inc., Research Triangle Park, NC 27709, USA; 5 Talecris Biotherapeutics GmbH, Lyoner Strasse 15, D-60528 Frankfurt am Main, Germany; 6 Lung Investigation Unit, University Hospitals of Birmingham, Edgbaston, Birmingham B15 2TH, UK

## Abstract

**Background:**

Computed tomography (CT) lung densitometry has been demonstrated to be the most sensitive and specific outcome measure for the assessment of emphysema-modifying therapy, but the optimum densitometric index has yet to be determined and targeted sampling may be more sensitive than whole lung assessment. The EXAcerbations and CT scan as Lung Endpoints (EXACTLE) trial aimed to clarify the optimum approach to the use of CT densitometry data for the assessment of alpha 1-antitrypsin (AAT) augmentation therapy on the progression of emphysema in AAT deficiency (AATD).

**Methods:**

Patients with AATD (n = 77) were randomised to weekly infusions of 60 mg/kg human AAT (Prolastin^®^) or placebo over 2 to 2.5 years. Lung volume was included as a covariate in an endpoint analysis and a comparison was made of different CT densitometric indices (15th percentile lung density [PD15], mean lung density [MLD] and voxel index at a threshold of -910 [VI-910] and -950 [VI-950] Hounsfield Units) obtained from whole lung scans at baseline and at 24 to 30 months. Targeted regional sampling was compared with whole lung assessment.

**Results:**

Whole lung analysis of the total change (baseline to last CT scan) compared with placebo indicated a concordant trend that was suggestive of a treatment effect for all densitometric indices (MLD [1.402 g/L, p = 0.204]; VI-910 [-0.611, p = 0.389]; VI-950 [-0.432, p = 0.452]) and that was significant using PD15 (1.472 g/L, p = 0.049). Assessment of the progression of emphysema in the apical, middle and basal regions of the lung by measurement with PD15 showed that this treatment effect was more evident when the basal third was sampled (1.722 g/L, p = 0.040). A comparison between different densitometric indices indicated that the influence of inspiratory variability between scans was greatest for PD15, but when adjustment for lung volume was made this index was the most sensitive measure of emphysema progression.

**Conclusion:**

PD15 is the most sensitive index of emphysema progression and of treatment modification. Targeted sampling may be more sensitive than whole lung analysis.

**Trial registration:**

Registered in ClinicalTrials.gov as 'Antitrypsin (AAT) to Treat Emphysema in AAT-Deficient Patients'; ClinicalTrials.gov Identifier: NCT00263887.

## Background

Computed tomographic (CT) imaging is the most sensitive and specific method for diagnosis of emphysema *in vivo *[1,2]. In addition, it provides quantitative data that correlate with pathological morphometry [3-6] and has been shown to be a valid tool for monitoring emphysema in clinical studies of alpha 1-antitrypsin deficiency (AATD) [7,8]. In recent years, there has been greater understanding and acceptance of this relatively novel technique, but there is limited published evidence to support the contention that one methodological approach to CT densitometry is superior to another. In particular, the majority of data have been obtained from observational cohorts [7-12], and it cannot be assumed that the conclusions of these studies may be extrapolated to interventional trials.

The EXACTLE (EXAcerbations and CT scan as Lung Endpoints) trial [13] was undertaken to explore the role of CT densitometry as a potential primary outcome measure in the setting of a double-blind, placebo-controlled study of the effect of alpha 1-antitrypsin (AAT) augmentation therapy on the progression of emphysema in individuals with AATD (PiZ) over 24 to 30 months. The study concluded that CT densitometry was a more sensitive and robust outcome measure than physiology, health status and exacerbation frequency, and demonstrated that the method for controlling the variability arising from differences in inspiratory level was of importance in demonstrating a treatment effect [10,13].

Additional CT methodological issues were explored in the EXACTLE study and the findings are reported here. These included the identification of the most discriminating densitometric index for use as an outcome measure. Furthermore, the role of regional densitometry was compared with whole lung densitometric assessment in order to determine whether targeted sampling was more appropriate for a pathological process that may be localised, and whether there could be regional differences in treatment effect.

## Methods

### Subjects

Patients with pulmonary emphysema due to severe congenital AATD of phenotype PiZ were recruited from AAT registries in Denmark, the UK and Sweden. Eligible patients were at least 18 years of age, had a history of at least 1 exacerbation in the past 2 years, had a post-bronchodilator forced expiratory volume in 1 second (FEV_1_) ≥ 25% and ≤ 80% predicted and a ratio of post-bronchodilator FEV_1 _to slow vital capacity (VC) ≤ 0.70, or a carbon monoxide transfer coefficient (DL_CO_/V_A_) of ≤ 80% of the predicted value, as previously reported [13]. All patients gave written informed consent. The study was approved by relevant local ethics review committees and was conducted in accordance with the Declaration of Helsinki and Good Clinical Practice guidelines.

### Study design

This multicentre, randomised, placebo-controlled, double-blind, parallel-group study was conducted at 3 centres in Copenhagen (Denmark), Birmingham (UK) and Malmö (Sweden). Eligible patients were randomly assigned, in permuted blocks with stratification according to country, to weekly infusions of *either *AAT (Prolastin^® ^60 mg/kg body weight) *or *placebo (2% concentration of albumin) for 24 months, as previously described [13]. CT scans were performed at baseline and at 12 and 24 months, with an option for continuation and an additional scan at 30 months.

### CT densitometry

**Figure 1:**
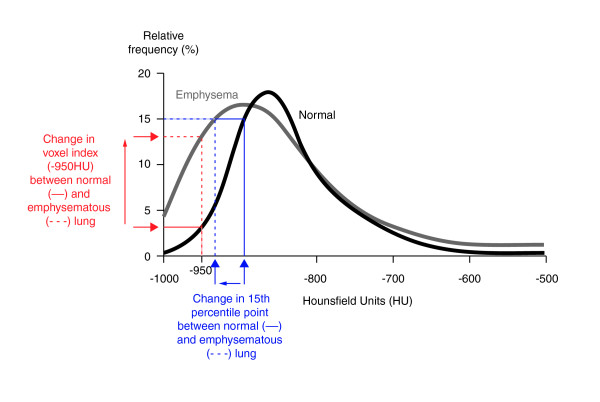
**Voxel frequency distribution histogram indicating the appearance in normal lung and in emphysema, and the derivation of the densitometric indices that were used in the current study**. 15th percentile point is defined as the cut-off value, in Hounsfield Units (HU), below which are distributed the 15% of voxels with the lowest density. (This index may be converted to the 15th percentile lung density (PD15) in g/L by the addition of 1000.) The voxel index at a threshold of -950HU (VI-950) is shown and is defined as the percentage of voxels with a value less than -950HU. The mean lung density is defined as the mean value (in g/L) of all voxels distributed within the lung histogram.

The primary efficacy endpoint was progression of emphysema determined by change in lung density measured by CT scan of the whole lung as previously reported [13]. Based on earlier studies [7,10,11,14] and recommendations of an expert review [15], the 15th percentile point was chosen as the parameter for the primary endpoint and expressed as the 15th percentile lung density (PD15). The 15th percentile point is defined as the value (Hounsfield Units) below which the 15% of voxels with the lowest density are distributed (Figure 1), and it may be expressed as PD15 (g/L) by the simple addition of 1000 to the Hounsfield value of the 15th percentile point. Other efficacy endpoints included the following additional densitometric indices extracted from the frequency distribution histogram of lung voxels: mean lung density (MLD), voxel index at a threshold of -910 Hounsfield Units (VI-910), and voxel index at a threshold of -950 Hounsfield Units (VI-950; Figure 1).

#### CT image acquisition

Multidetector CT scans of the chest were performed following inhaled bronchodilator therapy in the supine position during a breath-hold manoeuvre as close as possible to total lung capacity. All scanning was performed without intra-venous contrast in a caudo-cranial direction and with the arms held above the head in order to reduce artifacts. Scan acquisition parameters were standardized using the preferred scanning parameters 140 kVp, 40 mA, and pitch 1.5, with reconstructed slice thickness of 5 mm and with an increment of 2.5 mm but taking account of the scanner differences that existed between the 3 centres [13 and associated supplementary information]. Radiation per CT scan was low at around 1 mSv.

Mandatory scanner air calibration was performed according to the scanner manufacturers' instructions within 3 hours of the first patient scan, and every 3 hours during scanning lists. Mandatory water calibration was performed by the manufacturers (using the manufacturers' water phantom) at least every 3 months using the clinical scan protocol. Additional quality assurance was achieved using a dedicated Perspex and foam phantom that was scanned prior to site initiation, the first patient scan at each site, and every 6 months throughout the study.

Raw data were reconstructed using an edge-smoothing image reconstruction algorithm and were saved in DICOM format on CD for shipment to a central facility (Heart Core Global Medical Imaging) for densitometric analysis using dedicated software (Pulmo-CMS) as previously described [13 and associated supplementary information].

#### CT endpoints

Prior to un-blinding, a review panel assessed CT scan data to identify invalid scans due to technical issues [13 and associated supplementary information]. The progression of densitometric indices was estimated using an endpoint analysis using the first and last CT scans and incorporating adjustment for lung volume, as described below (see Statistical Analysis).

Progression was assessed for whole lung and for the apical, middle and basal regions. Subdivision of a whole lung series into apical, middle and basal thirds of approximate equal volume was achieved by dividing the whole lung into 12 segments of equal volume. The most apical and basal segments were excluded from further analysis because image artifact is recognised to be greatest in these anatomical locations [16]. The remaining 10 segments were divided into the 'basal region' (the 4 most caudal segments), the 'apical region' (the 3 most cranial segments) and the 'middle region' (the intervening 3 segments).

### Statistical analysis

All CT scan analyses were based on the modified intent-to-treat (mITT) population, defined as all randomised subjects who received the study therapy and had at least 1 valid CT scan measurement at baseline and 1 valid CT scan assessment at Month 12 or thereafter [13].

Treatment differences (Prolastin versus placebo) were tested using an analysis of covariance approach, with the change from baseline to the last CT scan measurement in lung density as the dependent variable, treatment and centre as fixed factors, and change in logarithm of CT-measured TLV and baseline measurement as covariates, as previously described [10,13]. This statistical model, in which lung volume attained during scan acquisition was log-transformed before it was used as a covariate, was applied to the analyses for all of the different densitometric indices. In contrast to the case when PD15 is used as the densitometric index, where log-transformed TLV is routinely used as a covariate in the statistical model, the optimum lung volume adjustment method for the voxel index parameters has not been established. Consequently, in the absence of any alternative, the same volume adjustment method was used for the voxel index as for PD15 in this study.

Sensitivity ratios were determined for each of the densitometric indices by dividing the value for the mean change from baseline in lung density by the standard error to obtain a sensitivity index. Sensitivity ratios measured by PD15 were also determined for the 3 lung regions. These data were obtained from analysis of the placebo group only. In order to establish the influence of inspiratory level on the different densitometric indices, additional sensitivity measurements were carried out in a post-hoc analysis without the lung volume adjustment.

## Results

### Patient characteristics at baseline

In total, of the 82 patients enrolled into the study from the 3 centres, 77 patients were randomised to Prolastin (n = 38) or placebo (n = 39), and 71 patients (n = 36, Prolastin; n = 35, placebo) were included in the mITT population. The number of patients in the ITT population who completed the study was 67, and 10 patients (3 in the Prolastin group and 7 in the placebo group) discontinued prematurely, resulting in a median of 127 weeks of exposure to Prolastin and 108 weeks to placebo. The study was completed by 92% of patients in the Prolastin group and 82% of patients in the placebo group (ITT population), as described previously [13].

Demographics and disease severity at baseline for all randomised patients are summarised in Table 1. Complete descriptive details have been previously reported [13]. CT data indicate that the majority of patients had predominantly basal emphysema and that there were no significant differences in lung density between the Prolastin and placebo groups at baseline (Table 1).

**Table 1:** Patient characteristics at baseline (ITT population)

	Prolastin (n = 38)	Placebo (n = 39)	p value
Age (years, mean ± SD)	54.7 ± 8.4	55.3 ± 9.8	0.749
Gender (n, male/female)	25/13	16/23	0.021
FEV_1_% predicted (mean ± SD)	46.3 ± 19.6	46.6 ± 21.0	0.873
PD15 (g/L, mean ± SD)^a^			
Whole lung	47.98 ± 19.07	45.48 ± 16.95	0.288
Apical region	63.36 ± 24.96	62.96 ± 19.77	0.625
Middle region	51.48 ± 18.35	48.35 ± 18.40	0.232
Basal region	38.61 ± 18.90	34.94 ± 15.44	0.182
MLD (g/L, mean ± SD)^a^	131.57 ± 21.99	131.68 ± 18.89	0.719
VI-910 (%, mean ± SD)^a^	44.43 ± 13.66	45.11 ± 12.02	0.509
VI-950 (%, mean ± SD)^a^	18.66 ± 10.97	19.77 ± 9.81	0.435
Lung weight (g, mean ± SD)^a^	956.40 ± 140.64	946.09 ± 224.12	0.750
Lung volume (L, mean ± SD)^a^	7.46 ± 1.60	7.27 ± 1.78	0.557

SD, standard deviation; FEV_1_, forced expiratory volume in 1 second; PD15, 15th percentile lung density; MLD, mean lung density; VI-910, voxel index at a threshold of -910 (measured in %); VI-950, voxel index at a threshold of -950 (measured in %). ^a^For the CT densitometric analyses, the mITT population was used (n = 36, Prolastin; n = 35, placebo).

### CT densitometric progression

All CT scan data were reviewed prior to study analysis in a blinded fashion to identify densitometric values that might be invalid because of technical issues, as previously described [13]. A total of 15 scans were invalid, which resulted in 6 patients having only 1 CT scan; these patients were therefore excluded from the mITT population.

#### Comparison of densitometric indices

The mean decline in lung density was determined and adjusted for lung volume, as described above (see Methods). All CT densitometric indices demonstrated a significant decline in both the Prolastin and placebo groups over the course of the study, consistent with emphysema progression (Table 2). The changes in PD15 from baseline to last CT scan were -2.645 g/L (Prolastin group) and -4.117 g/L (placebo group), indicating a significant treatment effect (p = 0.049) (Table 2). A trend towards a slower rate of decline in the Prolastin group was indicated when progression was assessed using MLD, VI-910 and VI-950, although the difference between the 2 treatment groups did not achieve statistical significance (p = 0.204, p = 0.389 and p = 0.452, respectively; Table 2).

**Table 2:** Comparison between different densitometric parameters (whole lung CT scans) to assess progression of emphysema in patients treated with Prolastin versus placebo (mITT population)

PD15 (g/L)	Prolastin (n = 36)	Placebo (n = 35)
Change from baseline to last CT scan (mean ± SD)	-2.895 ± 4.739	-4.124 ± 4.147
Change from baseline to last CT scan (LS mean [SE])	-2.645 (0.526) < 0.0001^a^	-4.117 (0.539) < 0.0001^a^
Estimated treatment difference between LS mean changes from baseline (95% CI)	1.472 (0.009, 2.935)	
p value for treatment difference^b^	0.049	

MLD (g/L)	Prolastin (n = 36)	Placebo (n = 35)

Change from baseline to last CT scan (mean ± SD)	-2.115 ± 7.937	-3.289 ± 5.949
Change from baseline to last CT scan (LS mean [SE])	-1.911 (0.788) 0.0181^a^	-3.313 (0.801) 0.0001^a^
Estimated treatment difference between LS mean changes from baseline (95% CI)	1.402 (-0.782, 3.586)	
p value for treatment difference^b^	0.204	

VI-910 (%)	Prolastin (n = 36)	Placebo (n = 35)

Change from baseline to last CT scan (mean ± SD)	1.761 ± 4.511	2.209 ± 3.378
Change from baseline to last CT scan (LS mean [SE])	1.643 (0.508) 0.0019^a^	2.254 (0.517) < 0.0001^a^
Estimated treatment difference between LS mean changes from baseline (95% CI)	-0.611 (-2.019, 0.797)	
p value for treatment difference^b^	0.389	

VI-950 (%)	Prolastin (n = 36)	Placebo (n = 35)

Change from baseline to last CT scan (mean ± SD)	1.994 ± 3.307	2.315 ± 2.578
Change from baseline to last CT scan (LS mean [SE])	1.924 (0.411) < 0.0001^a^	2.356 (0.420) < 0.0001^a^
Estimated treatment difference between LS mean changes from baseline (95% CI)	-0.432 (-1.573, 0.709)	
p value for treatment difference^b^	0.452	

PD15, 15th percentile lung density; CT, computed tomography; SD, standard deviation; LS mean, least squares mean; SE, standard error; 95% CI, 95% confidence interval; MLD, mean lung density; VI-910, voxel index at a threshold of -910 (measured in %); VI-950, voxel index at a threshold of -950 (measured in %). ^a^p values are for the comparison of change from baseline to last CT scan versus no change from baseline within the individual treatment groups. ^b^Prolastin treatment minus placebo (LS mean change).

The sensitivity ratios (whole lung assessment) measured for each of the densitometric parameters are shown in Table 3. PD15 was observed to be the most sensitive measure of emphysema progression.

**Table 3:** Sensitivity ratios for the different CT densitometric parameters (whole lung; analysis of covariance model)^a^

	Model with lung volume adjustment			
	
Outcome measure	LS mean change from baseline	Standard error	Sensitivity index^b^	F-test^c^
PD15 (g/L)	-4.12	0.539	7.64	-
MLD (g/L)	-3.31	0.801	4.14	< 0.05
VI-910 (%)	2.25	0.517	4.36	< 0.05
VI-950 (%)	2.36	0.420	5.61	NS

	Model without lung volume adjustment			
	
Outcome measure	LS mean change from baseline	Standard error	Sensitivity index^b^	Reduction in sensitivity index^d^

PD15 (g/L)	-4.24	0.771	5.50	2.14
MLD (g/L)	-3.49	1.221	2.86	1.28
VI-910 (%)	2.34	0.687	3.41	0.95
VI-950 (%)	2.41	0.514	4.69	0.92

LS mean, least squares mean; PD15, 15th percentile lung density; MLD, mean lung density;

VI-910, voxel index at a threshold of -910 (measured in %); VI-950, voxel index at a threshold of -950 (measured in %); NS, not significant. ^a^Results based on estimates from endpoint analysis for placebo group only. ^b^Ratio of absolute value of mean change divided by standard error. ^c^Ratio of outcome measure as compared to PD15. ^d^Sensitivity index from model with lung volume adjustment minus sensitivity index from model without lung volume adjustment.

#### Regional densitometry

**Figure 2:**
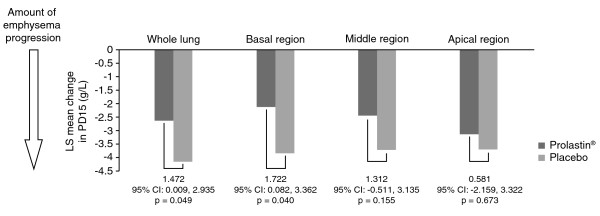
**Changes in PD15 (g/L) in whole lung and in basal, middle and apical regions in patients treated with Prolastin versus placebo (mITT population)**.

A significant decline in values for PD15 was observed in all 3 lung regions in both treatment groups during the study (Table 4). In the placebo arm, the rate of emphysema progression was comparable between the apical, middle and basal regions, whereas in the active treatment arm, the rate of emphysema progression in the basal region was lower than that of either the apical or middle regions. A significant treatment effect was demonstrated in the basal region (p = 0.040) and concordant trends were observed in the middle and apical regions, although these failed to achieve statistical significance (p = 0.155 and p = 0.673, respectively) (Table 4 and Figure 2). The sensitivity ratios showed that analysis by PD15 of the basal region was a significantly more sensitive measure than analysis of the apical region (Table 5).

**Table 4:** Changes in PD15 (g/L) in basal, middle and apical regions of the lung in patients treated with Prolastin versus placebo (mITT population)

Basal region	Prolastin (n = 36)	Placebo (n = 35)
Change from baseline to last CT scan (mean ± SD)	-2.336 ± 4.362	-3.760 ± 4.284
Change from baseline to last CT scan (LS mean [SE])	-2.118 (0.587) 0.0006^a^	-3.840 (0.604) < 0.0001^a^
Estimated treatment difference between LS mean changes from baseline (95% CI)	1.722 (0.082, 3.362)	
p value for treatment difference^b^	0.040	

Middle region	Prolastin (n = 36)	Placebo (n = 35)

Change from baseline to last CT scan (mean ± SD)	-2.845 ± 5.796	-3.838 ± 4.696
Change from baseline to last CT scan (LS mean [SE])	-2.504 (0.655) 0.0003^a^	-3.816 (0.673) < 0.0001^a^
Estimated treatment difference between LS mean changes from baseline (95% CI)	1.312 (-0.511, 3.135)	
p value for treatment difference^b^	0.155	

Apical region	Prolastin (n = 36)	Placebo (n = 35)

Change from baseline to last CT scan (mean ± SD)	-3.503 ± 7.433	-3.911 ± 5.939
Change from baseline to last CT scan (LS mean [SE])	-3.217 (0.990) 0.0018^a^	-3.799 (1.001) 0.0004^a^
Estimated treatment difference between LS mean changes from baseline (95% CI)	0.581 (-2.159, 3.322)	
p value for treatment difference^b^	0.673	

PD15, 15th percentile lung density; CT, computed tomography; SD, standard deviation; LS mean, least squares mean; SE, standard error; 95% CI, 95% confidence interval. ^a^p values are for the comparison of change from baseline to last CT scan versus no change from baseline within the individual treatment groups. ^b^Prolastin treatment minus placebo (LS mean change).

**Table 5:** Sensitivity ratios for the different lung regions (analysis of covariance model)^a^

	PD15 (g/L)				
	
Outcome measure	LS mean change from baseline	Standard error	Sensitivity index^b^	F-test^c^	F-test^d^
Whole lung	-4.12	0.539	7.64	-	-
Basal region	-3.84	0.604	6.36	NS	-
Middle region	-3.82	0.673	5.68	NS	0.642
Apical region	-3.80	1.001	3.80	< 0.05	< 0.05

PD15, 15th percentile lung density; LS mean, least squares mean; NS, not significant.^a^Results based on estimates for placebo group only. ^b^Ratio of absolute value of mean change divided by standard error. ^c^Ratio of outcome measure as compared to PD15 (whole lung). ^d^Ratio of outcome measure as compared to PD15 (basal region).

#### Effect of inspiratory level

A predefined correction for differences in inspiratory level between scans was applied as described in the Methods section, and a post-hoc investigation indicated a differential effect of this adjustment between the densitometric indices that was greatest for PD15 (Table 3).

## Discussion

The EXACTLE trial was designed to explore the use of CT densitometry as an outcome measure for the assessment of plasma AAT augmentation therapy in individuals with AATD. The analytical approach, and the principal technical issues that were addressed, were a logical sequence to previous studies within this field [7-12,14,17-21] and were integral to the design and statistical analysis plan of the EXACTLE trial [13]. The primary endpoint was the difference in lung density decline as a result of treatment, assessed from whole lung CT imaging and expressed as PD15, as reported previously [13].

### Densitometric indices

The current study included a comparison of the more commonly used densitometric indices to identify whether the advantages of the 15th percentile method, which have been demonstrated in observational studies, would also be evident in an interventional study. Previous studies have validated the use of several densitometric indices for the measurement of emphysema [4-6,22] and, although the 15th percentile method has not been compared directly with a pathological standard, clinical studies [7,10,14] support the theoretical advantages of this method [23] over the use of other indices. The sensitivity of the voxel index method has been shown in longitudinal studies to be influenced by the voxel index threshold [12,14] and by the severity of emphysema [7]. In contrast, the sensitivity of the percentile method is relatively independent of the chosen centile [14] and is a more consistent measure of emphysema progression across a wide spectrum of disease severity [7]. Furthermore, the correlation between the rate of reduction in lung density and the decline in FEV_1 _has been shown to be greater when the 15th percentile method is used [7]. This may reflect the better sensitivity of this measure of emphysema progression than the voxel index at a threshold of -950 and -910HU [10]. In the current study, a determination of the sensitivity ratios for the different densitometric indices confirms that the 15th percentile method is a more sensitive measure of emphysema progression than the voxel index method.

The results of the present study indicate that, following the adjustment of density values to correct for differences in inspiratory level [10,13], a significant decline in lung density is evident using all of the indices included, consistent with emphysema progression. A trend suggestive of a treatment effect was demonstrated for all indices and was statistically significant when lung density was assessed using PD15, as previously reported [13]. These findings confirm the results of previous studies [7,10,14] and provide yet further data that endorse the principle for using the percentile density method rather than the voxel index for monitoring studies [23], supporting the views of an expert panel [15].

However, notwithstanding this finding, the data indicate that differences in inspiratory level have a greater influence on PD15 than on the other indices included in the current study. Consequently, the incorporation of a method for correcting differences in lung volume between scans is more critical when PD15 is used.

### Regional densitometry

The rate of emphysema progression and treatment effect in different regions of the lung were also assessed by the 15th percentile method in the current study. It is of interest that, although the majority of subjects were shown to have predominantly basal emphysema at baseline, statistically significant decline in lung density was demonstrated in all 3 lung regions consistent with the progression of emphysema throughout the lung, as previously shown in an observational study [10]. The progression of emphysema identified in the placebo arm was similar in all lung regions and consistently greater than that seen in the treatment arm. However, this difference was only statistically significant in the basal region (p = 0.040), and targeted densitometric sampling of the basal region was shown to be more discriminative of a treatment effect than whole lung assessment. In addition, a significantly lower sensitivity index was obtained for PD15 assessment of the apical region compared with the basal region.

These data are of critical importance; not only do they provide information on the natural history of emphysema progression, but they may also influence the design and interpretation of future studies. Emphysema is understood to be a slowly progressive condition characterised by the development of specific patterns of disease distribution. These distribution patterns are viewed as pathognomonic of pathological sub-type and, as more recently implicated, of predisposing genotype [24,25]. Centrilobular emphysema is the most frequent pathological type that occurs in subjects with usual chronic obstructive pulmonary disease and is typically located towards the apical region [26,27], whereas the most common pathological type in subjects with AATD is panlobular emphysema, which is typically distributed in the basal region [16,27,28]. Although this is likely an oversimplification, and the 2 principal pathological types may co-exist [29,30], the pattern of emphysema distribution in the early stages of disease conforms to this description in the majority of individuals. As the disease progresses, it is likely that the extension of emphysema from these initial sites into unaffected areas will occur in a predictable sequence until, in severe disease, it becomes increasingly difficult to identify the initial pattern of distribution and the pathological sub-type [16].

There is no evidence to date that indicates a spatial differential susceptibility to the development and progression of emphysema within an individual lung, but no explanation has been offered to account for the localised development of emphysema that leads to the patterns of disease distribution described above. However, it is logical that the sites of initial disease must represent areas of the lung with increased potential susceptibility to emphysematous damage, since these areas are seemingly affected many years in advance of the remaining lung, and this pattern appears consistent across different patient populations. The current study supports the contention that there may be subtle differences in the pathogenesis of emphysema according to regional location within the lung, since the data clearly indicate a graded response to therapeutic augmentation of AAT. The graded therapeutic effect that was most evident in the basal region may indicate that the progression of panlobular emphysema might be retarded to a greater extent than the progression of centrilobular emphysema, since these 2 pathological sub-types are typically polarised towards the basal and apical regions, respectively. Unfortunately, it was not possible to perform a visual classification of emphysema sub-type in our cohort because the CT protocol that was used for the current study was intended for densitometric fidelity rather than optimum spatial resolution. However, previous descriptive studies have shown that apical centrilobular emphysema is evident in approximately half of subjects with PiZ AATD [30]. Whilst the above explanation for the data seems most plausible, alternative explanations may be proposed. For example, the tissue concentration of AAT may be greater in the basal region, since improved drug delivery would be anticipated by the greater pulmonary blood flow that is understood to exist in this region. Nevertheless, future studies will be required to address these issues.

## Conclusion

We have confirmed that PD15 is the most discriminative densitometric index for use in studies of emphysema-modifying therapy. Emphysematous destruction of the lung is understood to be heterogeneous, and pathognomonic patterns of emphysema distribution have been described. However, the current study shows that emphysema progression, as assessed by densitometry, occurs consistently throughout the whole lung. Importantly, the rate of lung density decline was reduced by the intravenous augmentation of plasma with AAT when assessed using PD15, the most sensitive parameter. Furthermore, the greatest effect was evident in the classically involved basal region of the lung, and targeted sampling may therefore be more sensitive in detecting a benefit of treatment on emphysema progression than whole lung assessment.

## Competing interests

DP has been in receipt of non-commercial funding from Talecris, lecture fees from Talecris and has sat on advisory boards to Talecris. AD has in the past years received reimbursements, fees and funding from Bayer and Talecris who financed the randomised clinical trial of which the current study is a spin-off. EP has received a fee for participation in an advisory board from Talecris who financed the randomised clinical trial of which the current study is a spin-off. MW and CD are employees of Talecris. RS has been in receipt of non-commercial funding from Talecris and lecture fees from Talecris; advisory board input to Talecris, Baxter and Kamada.

## Authors' contributions

DP contributed to the design of the study, in the analysis and interpretation of data, and drafting of the manuscript. AD was responsible for the Danish arm of EXACTLE and reviewing/contributing to writing the manuscript. EP was responsible for the Swedish arm of EXACTLE and reviewing the manuscript. MW and CD participated in the design of the study, in the collection, analysis and interpretation of data (CD was the statistician for the study), in the writing of the manuscript, in the decision to submit the manuscript for publication. RS was responsible for the UK arm of EXACTLE and reviewing/contributing to writing the manuscript. All authors have read and approved the final manuscript.
